# Growth differentiation factor-15 as a modulator of bone and muscle metabolism

**DOI:** 10.3389/fendo.2022.948176

**Published:** 2022-10-17

**Authors:** Seok Woo Hong, Jeong-Hyun Kang

**Affiliations:** ^1^ Department of Orthopedic Surgery, Kangbuk Samsung Hospital, School of Medicine, Sungkyunkwan University, Seoul, South Korea; ^2^ Clinic of Oral Medicine and Orofacial Pain, Institute of Oral Health Science, School of Medicine, Ajou University, Suwon, South Korea

**Keywords:** GDF-15, osteoporosis, sarcopenia, grip power, TNF-α, inflammation

## Abstract

This study aims to clarify the potential role of growth differentiation factor-15 (GDF-15) as a myokine in bone metabolism and muscle function in females with osteoporosis. In total, 45 female participants (71.0 ± 8.5 years) with distal radius fractures were recruited. Participants were classified as healthy/osteopenic (n = 28) (CON) or osteoporotic (n = 17) (OP) according to their T-score from the areal bone mineral density (aBMD) of the femoral neck. Body mass index, upper arm and calf circumferences, and handgrip strength were assessed. Total hip, femoral neck, and lumbar spine aBMD was measured *via* dual-energy x-ray absorptiometry. The focal bone quality of the distal radius was evaluated *via* 3D reconstructed computed tomographic images. Serum levels of GDF-15, insulin-like growth factor-1, and inflammatory markers such as tumor necrosis factor-α (TNF-α), interleukin-6, and interleukin-1β (IL-1β), as well as the corresponding mRNA levels in the pronator quadratus muscle were determined. Participants in the OP group had higher serum GDF-15 levels than those in the CON group. The mRNA levels of GDF-15, IL-1β, and TNF-α in the pronator quadratus muscle were significantly higher in the OP group than in the CON one. Levels of both serum GDF-15 and GDF-15 mRNA in muscle were positively correlated with age and negatively associated with the aBMD of the total hip and focal bone quality of the distal radius. Handgrip power was not correlated with circulating GDF-15 levels but was correlated with circumferences of the upper arm and calf, and levels of GDF-15 mRNA in muscle specimens. The mRNA levels of GDF-15 were correlated with those of inflammatory cytokines such as TNF-α and IL-1β. The mRNA levels of TNF-α were associated with circumferences of the upper arm and calf and with the aBMD of the total hip. The mRNA levels of GDF-15 in muscle were correlated with serum levels of GDF-15 and TNF-α. GDF-15 may have associations with bone metabolism in humans *via* paracrinological and endocrinological mechanisms. Maintenance of muscle mass and function would be influenced more by GDF-15 in muscle than by circulating GDF-15. The role of GDF-15 in bone metabolism and muscle homeostasis could be related to inflammatory responses.

## Introduction

Aging is accompanied by changes in neuromuscular, immunological, and endocrinological functions that may result in decreased muscle and bone mass and frailty in elderly people (1). The concept of “sarco-osteopenia” was proposed to diagnose the coexistence of osteoporosis and sarcopenia (2). Skeletal muscle and bone are the two major components of the musculoskeletal system, and they are closely related mechanically through the lever and pulley system and also have bidirectional chemical interactions involving endocrinological and paracrinological mechanisms (3). Myokines are soluble molecules that are expressed and released by muscle fibers and regulate the biological and pathological activities of cells and organs (4). The various types of myokines that maintain homeostasis between muscle and bone and influence the bone remodeling process have been identified (5). The recent trends in therapeutic approaches to prevent osteoporosis and sarcopenia have focused on this muscle-bone interaction and myokines.

Growth differentiation factor-15 (GDF-15), a member of the transforming growth factor β (TGF-β) superfamily, has been implicated as a biomarker of aging, sarcopenia, muscle wasting, cachexia, mitochondrial disease, decreased physical activity, and energy imbalance (6–15). As a circulating myokine, the serum level of GDF-15 is increased as a result of a metabolic response to muscle-specific mitochondrial stress and muscle dysfunction in humans (9, 16, 17). Increased levels of GDF-15 in skeletal muscle affect metabolic homeostasis, improve insulin resistance, and protect against obesity by inducing lipolysis and oxidative metabolism (16–18). Hence, the importance of GDF-15 activity in maintaining the proper function and metabolic balance of skeletal muscles could be assumed.

Several previous studies revealed links between GDF-15 and the bone remodeling process, but the findings were contradictory. One study showed that GDF-15 was a positive secretory regulator of osteoclastic differentiation in osteocytes under conditions of hypoxia during bone remodeling (19). Another study demonstrated that GDF-15 plays a role in the functional modulation of osteoclast progenitors in response to compressive force (20). Yet another study showed that prostate adenocarcinoma Cell line treated with GDF-15 suppressed the formation of mature osteoclast differentiation in macrophages and bone marrow precursors (21). Furthermore, it was hypothesized that GDF-15 could control osteoclast activity and bone metastasis from osteosarcoma, lung cancer, and prostate cancer in animal models (22–24) and patients with multiple myeloma (25), but those reports also showed conflicting results. Even though the exact mechanisms between GDF-15 and bone remodeling have not been elucidated so far, GDF-15 does play a role in the bone remodeling process.

Previous studies have described the links among GDF-15, osteoclastic activity, and muscle metabolism. The precise role of GDF-15 in skeletal muscle in maintaining bone homeostasis and metabolism, however, remains unclear. To the best of our knowledge, few attempts have been made to reveal the exact role of GDF-15 as a myokine in skeletal muscles in bone-muscle interactions. Therefore, the aim of the current study was to clarify the potential role of GDF-15 as a myokine in bone metabolism and muscle function in patients with osteoporosis.

## Materials and methods

### Participants

In total, 45 Korean female participants (mean age 71.0 ± 8.5 years, age range 55–93 years) with distal radius fractures that required surgical treatment were recruited from a tertiary care hospital. All participants were post-menopausal. The participants were subsequently divided into healthy/osteopenic participants (n = 28) (CON) and osteoporotic participants (n = 17) (OP) based on their T-score from the bone mineral density in the femoral neck. An orthopedic surgeon treated all fractures with open reduction and internal fixation using a volar plate fixation system. A trained nurse measured the body mass index (BMI) and circumferences of the upper arm and calf. Participants with a history of taking bone active medications, including bisphosphonates, denosumab, and hormone modulating agents, as well as those with neurodegenerative diseases, rheumatoid arthritis, uncontrolled diabetes mellitus, heart failure, kidney failure, cancer, and thyroid disorder were excluded, as were those who were uncommunicative.

The research protocol was approved by the Institutional Review Board of the University Hospital (KBSMC-2020-07-016). Informed consent was obtained from all participants.

### Measurement of handgrip strength

Handgrip strength was measured by a hand dynamometer (Jamar^®^ 5030J1 hydraulic hand dynamometer, Sammons Preston Rolyan, Bolingbrook, IL, USA) with the non-injured contralateral hand by an orthopedic surgeon. The measurement was obtained while the patient sat with the non-injured elbow flexed to 90˚ and the forearm in a neutral position (26). All participants were instructed to perform a handgrip strength test with their maximum grip strength. Handgrip strength was measured three times for each participant, and the average value was subsequently calculated. If the dominant hand was on the injured side, the 10% rule was applied to estimate handgrip strength (27, 28).

### Evaluation of bone mineral density

The areal bone mineral densities (aBMDs, in grams per square centimeter) of the total hip, femoral neck, and lumbar spine (L1-L4) were assessed with a dual-energy x-ray absorptiometry (DEXA) with a Hologic device (Horizon-W; Hologic Inc., Bedford, MA, USA). The T-scores of the aBMDs of the total hip, femoral neck, and lumbar spine were evaluated based on the value of the aBMDs.

### Biochemical evaluation

Peripheral venous blood samples from each participant were collected between 8:00 and 11:00 a.m. after fasting overnight to minimize circadian rhythm variabilities. The serum levels of the C-telopeptide of type I collagen (CTx), osteocalcin, insulin-like growth factor-1 (IGF-1), and 25-hydroxyvitamin D were analyzed. Electrochemiluminescence immunoassays were used to measure the levels of CTx (Roche Diagnostics, Basel, Switzerland) and IGF-1 (Siemens Healthcare Diagnostics, NY, USA). A chemiluminescence immunoassay (Roche Diagnostics, Basel, Switzerland) was used to determine the concentration of osteocalcin. The concentration of serum 25-hydroxyvitamin D was assessed with an electrochemiluminescence binding assay (Roche Diagnostics, Basel, Switzerland).

To determine the serum levels of GDF-15, tumor necrosis factor-α (TNF-α), interleukin-6 (IL-6), and interleukin-1β (IL-1β), we used Quantikine ELISA kits (R&D Systems, Minneapolis, MN, USA). All assays were performed in triplicate, and the data were averaged. The intra- and inter-assay coefficients of variation of the GDF-15 assay were 1.8% and 4.7%, respectively, and its sensitivity was 4.39 pg/ml. Those for TNF-α were 2.2% and 7.3%, and its sensitivity was 6.23 pg/ml; those for IL-6 were 1.6% and 3.3%, and its sensitivity was 0.7 pg/ml, and; those for IL-1 β were 2.8% and 4.1%, and its sensitivity was 1.0 pg/ml.

### Muscle biopsies

An orthopedic surgeon harvested all muscles during the open reduction and internal fixation procedures of the distal radius fracture. While the patients were under general anesthesia, a 4 cm longitudinal incision was made along the radial border of the flexor carpi radialis (FCR) tendon. The FCR tendon was retracted ulnarly, and the dorsal aspect of the FCR sheath was incised to expose the flexor pollicis longus tendon. The flexor pollicis longus (FPL) tendon was also retracted ulnarly, and the pronator quadratus muscle was exposed. A small part of the pronator quadratus muscle (1 mm x 1 mm x 2 mm) was harvested from all participants during the elevation of the muscle from the distal radius bone. Immediately after harvesting, the muscle specimens were placed in a cryotube, frozen with liquid nitrogen, and stored at -80°C until analysis began.

### Real-time PCR analysis

Total RNA was extracted from the pronator quadratus muscle specimens using QIAZOL Lysis Reagent (QIAGEN, Hilden, Germany) with a homogenizer. Genomic DNA contamination was removed using RNase-free DNase I (QIAGEN, Hilden, Germany). The amount of total RNA was measured using NanoDrop 2000 (Thermo Fisher Scientific Invitrogen Inc., MA, USA). Real-time qPCR reactions were performed with an ABI 7500 (Applied Biosystem, Foster City, CA, USA) using an SYBR Green PCR kit (Takara Bio, Shiga, Japan), and cDNA was synthesized using 1000 μɡ of total RNA (Thermo Fisher Scientific Invitrogen Inc., MA, USA). Each sample was assessed in triplicate and normalized against the expression level of the housekeeping gene GAPDH. All primer sequences including those of GAPDH, GDF-15, IGF-1, and the inflammatory markers TNF-α, IL-6, and IL-1β are reported in **Table 1**.

**Table 1:** Sequence of each primer.

Primer	Forward primer	Reverse primer
GDF-15	CAATCCCATGGTGCTCATTC	TATGCAGTGGCAGTCTTTGG
IL-1β	GTACCTGTCCTGCGTGTTGA	GGGAACTGGGCAGACTCAAA
IL-6	CTATGcAACTCCTTCTCCACAAGCGCCTT	GGGGCGGCTACATCTTTGGAATCTT
TNF-α	CTTCTGCCTGCTGCACTTTG	GTCACTCGGGGTTCGAGAAG
IGF-1	CCATGTCCTCCTCGCATCTC	CGTGGCAGAGCTGGTGAAG
GAPDH	CCATCTTCCAGGAGCGAGATC	GCCTTCTCCATGGTGGTGAA

GDF-15, growth differentiation factor-15; IL-1β, interleukin-1beta; IL-6, interleukin-6; TNF-α, tumor necrosis factor-alpha; IGF-1, insulin-like growth factor-1; GAPDH, Glyceraldehyde-3-Phosphate-Dehydrogenase.

### Reconstruction of the 3D bone model and bone quality of distal radius

**Figure 1:**
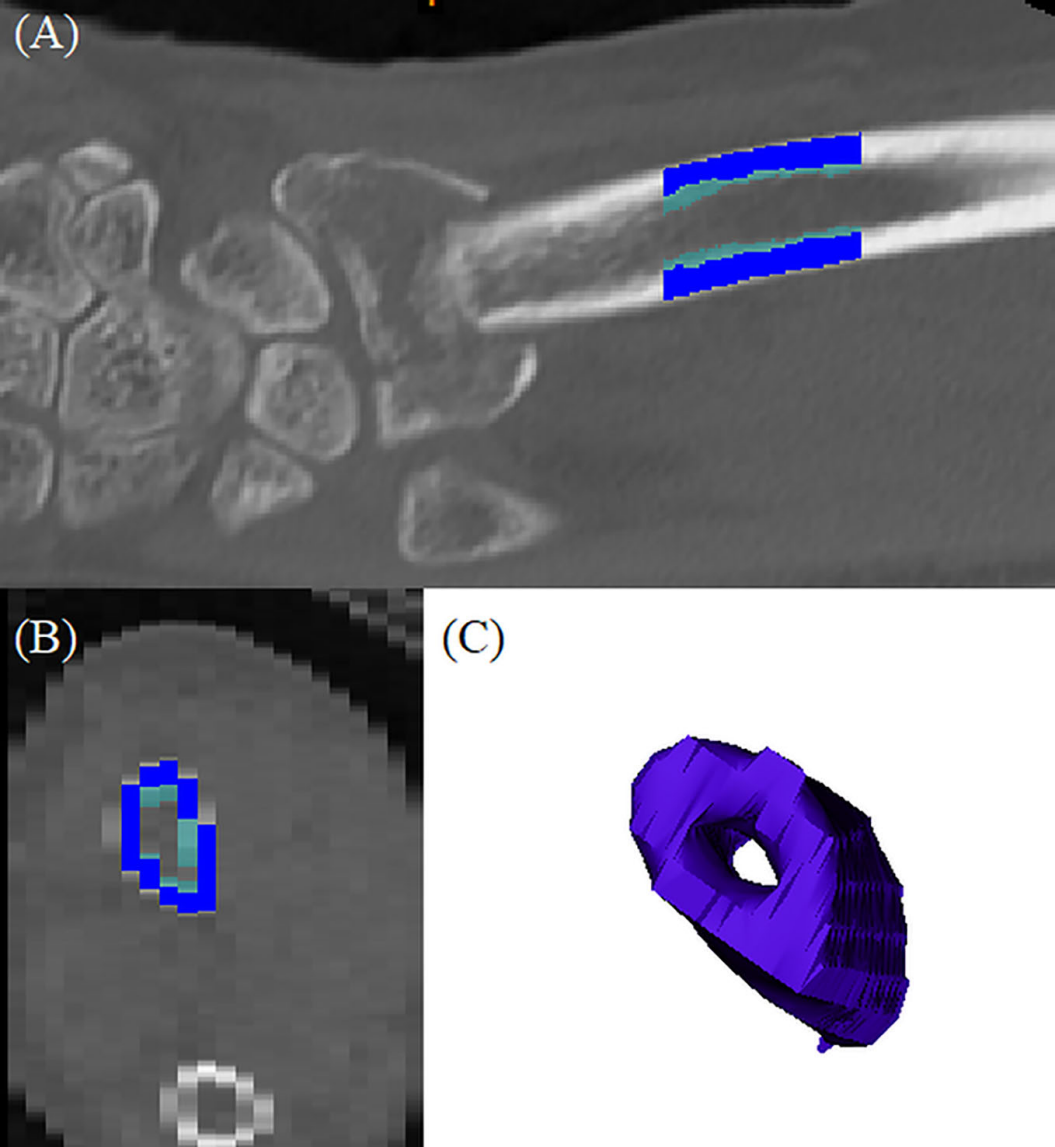
3D reconstructed distal radius bone model: **(A)** coronal section of the original CT images; dark blue indicated cortical bone and light blue indicated trabecular bone, **(B)** axial section of the original CT image; dark blue indicated cortical bone and light blue indicated trabecular bone, **(C)** reconstructed bone model.

Wrist CT images were obtained immediately after the closed reduction of the fracture at the emergency department and before open reduction surgery using a 256-slice multi-detector CT scanner (Brilliance iCT 256, Philips Medical Systems, Amsterdam, Netherlands). The scanning protocol described below was used: 120 kVp tube potential; 149 mAs tube current-time product; 128 mm × 0.625 mm section collimation; 0.5 ms rotation time; 0.4 pitch; 180 mm display field of view; pixel size 0.3 mm × 0.3 mm; and 1 mm section thickness. Corrected coronal, sagittal, and axial images of the wrist were saved as Digital Imaging and Communications in Medicine (DICOM) files. DICOM format files were imported into a 3D reconstruction modeling software (Mimics 22.0, Materialise, Antwerp, Belgium). A 2 cm long cylindrical bone model was constructed 3 cm proximal to the lunate fossa of the radius. A global threshold of 200 Hounsfield units (HU) was applied to the CT scans to obtain a representation of the 3D wrist bone model, and 850 HU was used to reconstruct the cortical bone structures. The average cortical and trabecular HU, total HU, and cortical thickness of the wrist bone models were calculated automatically using the Mimics 22.0^®^ software and 3-matic 14.0 ^®^ software (Materialise, Antwerp, Belgium) (29–31) (**Figure 1**). To assess inter-examiner reliability, one orthopedic surgeon and one orofacial pain specialist assessed 30 randomly selected CT data, and the analyses from each examiner were compared (inter-examiner) using intraclass correlation coefficient (ICC) to assess the reliability. The ICCs for average HU and cortical thickness were 0.786 and 0.763 with statistical significance, respectively. One examiner repeated the process on 10 randomly selected CT images after 2 weeks (intra-examiner), and the data were compared using ICC, and an acceptable agreement was found. The ICC was 0.816 with statistical significance.

### Statistical analysis

The Shapiro-Wilk normality test revealed that the data from the present study were normally distributed, and parametric tests were applied. In comparisons of the parameters of the CON and OP groups, the independent t-test and Chi-square test were used for continuous and categorical variables, respectively. To determine the relationships between the variables, Pearson’s correlation coefficient was applied. All values were considered significant when *P *< 0.05.

## Results

### Differences in DEXA score and local bone quality of the distal radius

The age (*P* = 0.144) between the two groups was not statistically different. On the other hand, BMI (*P* = 0.044), handgrip strength (*P* = 0.004), and the circumferences of the upper arm and calf (*P* = 0.047) showed significant differences between groups. The aBMD and T-scores of the total hip (*P* < 0.001), femoral neck (*P* < 0.001), and lumbar spine (*P* < 0.001) also demonstrated significant differences between groups, respectively. Significant differences were also found in the total HU (*P* < 0.001), cortical HU (*P* < 0.001), cortical thickness (*P* = 0.005), and trabecular HU (*P* < 0.001) of 3D reconstructed bone models of the distal radius. Participants in OP had significantly lower grip power, aBMD of the total hip, femoral neck, lumbar spine, and focal bone quality of the distal radius (**Table 2**).

**Table 2:** Differences of demographic features, BMD, and local radial forearm bone quality between two groups.

	CON (n = 28)	OP (n = 17)	*P*-value
Age (years)	69.5 ± 6.3	73.4 ± 11.0	0.144
Handgrip strength (kg)	20.1 ± 2.9	16.4 ± 5.3	0.004*
BMI	25.1 ± 3.0	23.2 ± 2.9	0.044*
Upper arm circumference	28.9 ± 3.3	26.2 ± 3.4	0.103
Calf circumference	33.3 ± 2.6	31.6 ± 3.1	0.047*
Total hip aBMD (g/cm^2^)	0.76 ± 0.07	0.62 ± 0.08	< 0.001**
Total hip T-score	-0.85 ± 0.70	-2.12 ± 0.61	< 0.001**
Femoral neck aBMD (g/cm^2^)	0.64 ± 0.08	0.50 ± 0.04	< 0.001**
Femoral neck T-score	-1.70 ± 0.52	-2.87 ± 0.39	< 0.001**
L1-4 aBMD (g/cm^2^)	0.84 ± 0.13	0.72 ± 0.12	0.004*
L1-4 T-score	-1.64 ± 1.17	-2.51 ± 0.96	0.010*
Total HU	958.6 ± 131.0	854.2 ± 118.0	< 0.001**
Cortical HU	1261.2 ± 131.3	1087.4 ± 147.0	< 0.001**
Cortical thickness (mm)	1.21 ± 0.40	0.90 ± 0.21	0.005*
Trabecular HU	481.8 ± 14.4	465.5 ± 14.1	< 0.001**

aBMD, areal bone mineral density; BMI, body mass index; L1-4, lumbar spine 1-4; HU, Hounsfield unit.

Descriptive values are shown as mean ± SD.

Data obtained from independent t-test.

*P < 0.05, **P < 0.001 by independent t-test and Chi-square test.

### Differences in serum levels of bone turnover markers, GDF-15, and inflammatory cytokines

The serum levels of CTx (*P* = 0.904), osteocalcin (*P* = 0.112), IGF-1 (*P* = 0.799), 25-hydroxyvitamin D (*P* = 0.788), TNF-α (*P* = 0.115), IL-6 (*P* = 0.203), and IL-1β (*P* = 0.459) did not show significant differences between CON and OP. Otherwise, the differences in the serum concentrations of GDF-15 were statistically significant (*P* = 0.019), and participants in OP had significantly higher levels of serum GDF-15 than those in CON (**Table 3**).

**Table 3:** Differences of serum levels of bone turnover markers, IGF-1, vitamin D, GDF-15, and inflammatory cytokines between CON and OP.

	CON (n = 28)	OP (n = 17)	*P*-value
CTx (ng/ml)	0.45 ± 0.28	0.47 ± 0.31	0.774
Osteocalcin (ng/ml)	17.6 ± 9.1	22.6 ± 9.8	0.071
IGF-1 (ng/ml)	106.3 ± 50.2	102.7 ± 59.9	0.819
25-hydroxyvitamin D (ng/ml)	25.4 ± 15.6	27.6 ± 15.5	0.630
GDF-15 (pg/ml)	831.0 ± 449.7	1167.0 ± 687.3	0.037*
TNF-α (pg/ml)	18.6 ± 7.4	41.7 ± 84.6	0.112
IL-6 (pg/ml)	13.8 ± 14.1	16.8 ± 20.0	0.521
IL-1 β (pg/ml)	0.51 ± 0.24	0.56 ± 0.35	0.512

CTx, C-telopeptide of type I collagen; IGF-1, insulin-like growth factor-1; GDF-15, growth differentiation factor-15, TNF-α, tumor necrosis factor-α; IL-6, interleukin-6; IL-1β, interleukin-1β.

Descriptive values are shown as mean ± SD.

Data obtained from independent t-test.

*P < 0.05 by independent t-test. CON, healthy/osteopenic participants based on their T-score from the bone mineral density in the femoral neck; OP, osteoporotic participants based on their T-score from the bone mineral density in the femoral neck.

### Expression levels of GDF-15 and inflammatory cytokines in pronator quadratus muscle specimens

**Figure 2:**
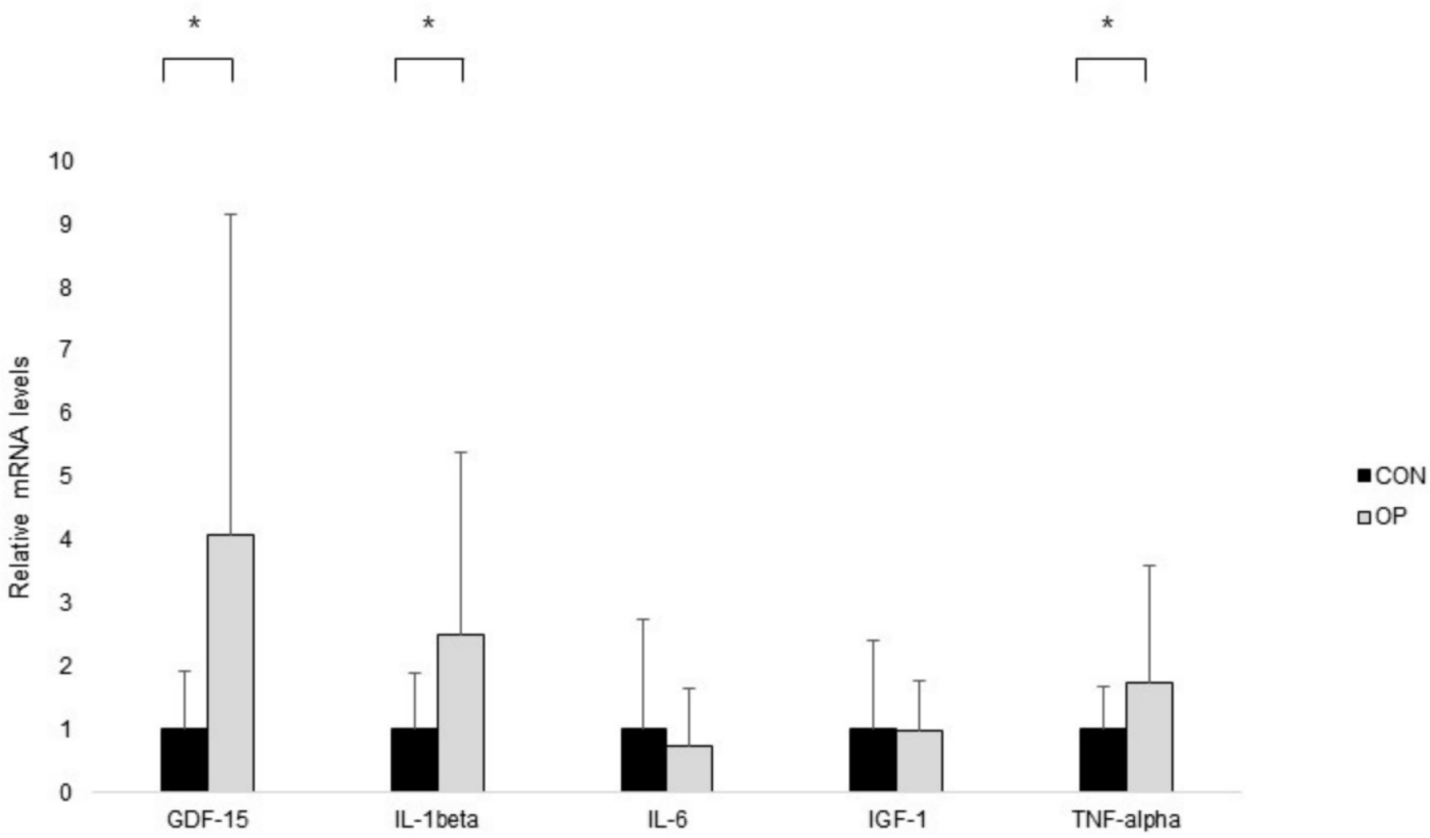
The results of real-time PCR. Normalized fold expressions of mRNA are shown. The error bar represents the mean ± standard error. To compare gene expression levels between groups, independent t-test was applied (*P < 0.05).

The significantly different levels of mRNA expression in the pronator quadratus muscle in GDF-15 (*P* = 0.003), IL-1β (*P* = 0.013), and TNF-α (*P* = 0.034) between the two groups were shown with those in the OP were significantly higher than those in the CON. Otherwise, there were no statistically significant differences expressed in the mRNA levels of IL-6 (*P* = 0.580) and IGF-1 (*P* = 0.955) between the groups (**Figure 2**).

### Correlations among age, body parameters, and skeletal BMD

The extents of muscle mass and power showed significant associations with skeletal BMD, and age was shown to have significantly negative impacts on skeletal aBMD and handgrip strength. Handgrip strength showed a significantly negative correlation with age (r^2^ = -0.579, *P* < 0.001) and a positive correlation with the circumferences of the upper arm and calf (r^2^ = 0.388, *P* = 0.009), aBMD of the total hip (r^2^ = 0.452, *P* < 0.001) and femoral neck (r^2^ = 0.459, *P* < 0.001), cortical HU (r^2^ = 0.348, *P* = 0.019), and cortical thickness (r^2^ = 0.315, *P* = 0.035) of the bone model of the distal radius. The focal bone density of the distal radius showed significant relationships with aBMD of the total hip, femoral neck, and lumbar spine. The circumferences of the upper arm and calf showed significantly positive associations with aBMD of the total hip (r^2^ = 0.413, *P* < 0.001), femoral neck (r^2^ = 0.329, *P* = 0.027), and trabecular HU of the distal radius bone (r^2^ = 0.347, *P* = 0.020), respectively (**Table 4**).

**Table 4:** Correlation coefficient among age, handgrip strength, BMI, circumferences of upper arm and calf, aBMD of total hip, femoral neck, and lumbar spine, and local bone quality of radial forearm.

	Age	Grip strength	BMI	Upper arm circumference	Calf circumference	Total hip aBMD	Femoral neck aBMD	Lumbar spine aBMD	Total HU	Cortical HU	Cortical thickness
Grip strength	-0.579^**^										
BMI	-0.035	0.022									
Upper arm circumference	-0.201	0.249	0.624^**^								
Calf circumference	-0.384^*^	0.388^*^	0.277	0.260							
Total hip aBMD	-0.338^*^	0.452^**^	0.246	0.237	0.413^**^						
Femoral neck aBMD	-0.201	0.459^**^	0.148	0.122	0.329^*^	0.733^**^					
Lumbar spine aBMD	-0.137	0.190	0.200	0.096	0.188	0.357^*^	0.400^**^				
Total HU	-0.364^*^	0.182	0.104	0.033	-0.016	0.306^*^	0.167	0.281			
Cortical HU	-0.300^*^	0.348^*^	0.144	0.148	0.185	0.462^**^	0.319^*^	0.323^*^	0.883^**^		
Cortical thickness	-0.141	0.315^*^	0.068	0.046	0.004	0.337^*^	0.214	0.185	0.701^**^	0.761^**^	
Trabecular HU	-0.302^*^	0.240	0.243	0.096	0.347^*^	0.432^**^	0.403^*^	0.418^**^	0.543^**^	0.654^**^	0.443^**^

BMI, body mass index; aBMD, areal bone mineral density; HU, Hounsfield units.

^*^P < 0.05, ^**^P < 0.001 by Pearson’s correlation analysis.

The figures in bold indicate values with statistical significance.

### Associations among serum levels of GDF-15, body parameters, skeletal BMD, and levels of inflammatory cytokines

The serum levels of GDF-15 showed significantly positive correlations with age (r^2^ = 0.374, *P* = 0.011). It had significantly negative associations with the aBMD of the total hip (r^2^ = -0.366, *P* = 0.013) and focal bone quality of the distal radius. Otherwise, the serum concentrations of GDF-15 did not show significant correlations with BMI (r^2^ = -0.234, *P* = 0.122), handgrip power (r^2^ = -0.227, *P* = 0.134), the circumferences of the upper arm (r^2^ = -0.213, *P* = 0.161) and calf (r^2^ = -0.219, *P* = 0.149), and the serum levels of IGF-1 (r^2^ = -0.125, *P* = 0.412) and inflammatory cytokines such as TNF-α (r^2^ = 0.181, *P* = 0.235), IL-6 (r^2^ = -0.073, *P* = 0.631), and IL-1β (r^2^ = 0.161, *P* = 0.289). The serum concentration of TNF-α showed negative correlations with handgrip strength (r^2^ = -0.536, *P* < 0.001) and the circumferences of the upper arm and calf (r^2^ = -0.438, *P* < 0.001). Circulating levels of GDF-15, TNF-α, IL-6, and IL-1β showed significant relationships with focal density for the distal radius (**Table 5**). When correlations were analyzed in the CON and OP groups separately, GDF-15 showed a significant association with the aBMD of the total hip, and cortical and trabecular bone density of the distal radius bone were only significant in OP not in CON (**Supplementary Figure**).

**Table 5:** Relationships among age, handgrip strength, BMI, circumferences of upper arm and calf, serum GDF-15 and cytokine levels, handgrip strength, aBMD of total hip, femoral neck, and lumbar spine, and local bone quality of radial forearm.

	Serum GDF-15	Serum TNF-α	Serum IL-6	Serum IL-1 β	Serum IGF-1
Age	0.374^*^	0.423^**^	0.150	-0.071	-0.247
Grip strength	-0.227	-0.536^**^	-0.190	0.040	0.152
BMI	-0.234	-0.105	0.063	-0.001	0.235
Upper arm circumference	-0.213	-0.219	0.033	-0.049	0.330^*^
Calf circumference	-0.219	-0.438^**^	-0.068	0.070	0.247
Total hip aBMD	-0.366^*^	-0.291	-0.370^*^	-0.103	0.212
Femoral neck aBMD	-0.232	-0.191	-0.335^*^	-0.184	-0.127
Lumbar spine aBMD	-0.152	-0.128	-0.239	-0.168	0.032
Total HU	-0.248	-0.125	-0.319^*^	-0.139	-0.036
Cortical HU	-0.354^*^	-0.317^*^	-0.357^*^	-0.279	0.012
Cortical thickness	-0.132	-0.133	-0.406^**^	-0.159	-0.130
Trabecular HU	-0.373^*^	-0.084	-0.279	-0.305^*^	0.147
Serum GDF-15		0.181	-0.073	0.161	-0.125
Serum TNF-α			0.035	-0.165	-0.132
Serum IL-6				0.438^**^	-0.094
Serum IL-1 β					-0.084

BMI, body mass index; aBMD, areal bone mineral density; HU, Hounsfield units

^*^P < 0.05, ^**^P < 0.001 by Pearson’s correlation analysis

The figures in bold indicate values with statistical significance.

GDF-15, growth differentiation factor-15; IL-1β, interleukin-1beta; IL-6, interleukin-6; TNF-α, tumor necrosis factor-alpha; IGF-1, insulin-like growth factor-1.

### Associations among mRNA expression levels of GDF-15 and inflammatory cytokines in pronator quadratus muscle specimens, body parameters, and skeletal BMD

The mRNA expression levels of GDF-15 in muscle specimens were significantly associated with age (r^2^ = 0.404, *P* < 0.001). Muscle specimens had significantly negative relationships with handgrip strength (r^2^ = -0.515, *P* < 0.001), circumferences of the upper arm (r^2^ = -0.378, *P* = 0.011) and calf (r^2^ = -0.443, *P* < 0.001), aBMD of the total hip (r^2^ = -0.392, *P* = 0.008), and cortical HU (r^2^ = -0.480, *P* < 0.001) and trabecular HU (r^2^ = -0.374, *P* = 0.011) of the distal radius. Furthermore, it correlated significantly with expression levels of inflammatory cytokines such as TNF-α (r^2^ = 0.643, *P* < 0.001) and IL-1β (r^2^ = 0.909, *P* < 0.001). The mRNA expression levels of TNF-α significantly negatively associated with circumferences of upper arm (r^2^ = -0.309, *P* = 0.044) and calf (r^2^ = -0.314, *P* = 0.035), aBMD of the total hip (r^2^ = -0.426, *P* < 0.001), and trabecular HU (r^2^ = -0.351, *P* = 0.018) of the distal radius. The expression levels of GDF-15 correlated significantly with serum GDF-15 (r^2^ = 0.564, *P* < 0.001) and TNF-α levels (r^2^ = 0.694, *P* < 0.001) (**Table 6**). When the correlation analysis was conducted separately in CON and OP, only in OP did the mRNA levels of GDF-15 in muscle show significant relationships with age, grip strength, circumferences of upper arm and calf, and cortical and trabecular bone density of the distal radius bone. The relationship between mRNA levels of GDF-15 and those of TNF-α showed significant correlations in both CON and OP (**Supplementary Figure**).

**Table 6:** Correlation among age, handgrip strength, BMI, circumferences of upper arm and calf, mRNA expression levels of GDF-15 and cytokine in muscles, aBMD of total hip, femoral neck, and lumbar spine, and local bone quality of radial forearm.

	GDF-15	TNF-α	IL-6	IL-1 β	IGF-1
Age	0.404^**^	0.242	0.074	0.418^**^	0.212
Grip strength	-0.515^**^	-0.192	0.114	-0.447^**^	-0.177
BMI	-0.182	-0.182	-0.214	-0.238	-0.179
Serum GDF-15	0.564^**^	0.486^**^	0.202	0.511^**^	0.077
Serum TNF-α	0.694^**^	0.336^*^	-0.105	0.625^**^	0.017
Serum IL-6	0.033	-0.056	-0.087	-0.065	-0.099
Serum IL-1 β	0.125	0.075	0.058	0.062	-0.112
Serum IGF-1	-0.144	-0.051	-0.128	-0.186	-0.102
Upper arm circumference	-0.378^*^	-0.309^*^	0.016	-0.366^*^	-0.235
Calf circumference	-0.443^**^	-0.314^*^	-0.225	-0.441^**^	-0.292
Total hip aBMD	-0.392^*^	-0.426^**^	0.067	-0.291	-0.133
Femoral neck aBMD	-0.248	-0.267	0.044	-0.198	0.043
Lumbar spine aBMD	-0.276^*^	-0.290	-0.031	-0.216	0.030
Total HU	-0.209	-0.050	-0.078	-0.163	0.016
Cortical HU	-0.480^**^	-0.284	-0.056	-0.402^**^	0.030
Cortical thickness	-0.286	-0.213	-0.083	-0.229	0.012
Trabecular HU	-0.374^*^	-0.351^*^	-0.272	-0.396^*^	0.026
GDF-15		0.643^**^	-0.080	0.909^**^	0.140
TNF-α			0.001	0.639^**^	0.160
IL-6				0.162	0.140
IL-1 β					0.140

BMI, body mass index; aBMD, areal bone mineral density; HU, Hounsfield units

*P < 0.05, ^**^P < 0.001 by Pearson’s correlation analysis

The figures in bold indicate values with statistical significance.

GDF-15, growth differentiation factor-15; IL-1β, interleukin-1beta; IL-6, interleukin-6; TNF-α, tumor necrosis factor-α; IGF-1, insulin-like growth factor-1.

## Discussion

GDF-15, a member of TGF-β superfamily, has been identified as a marker of aging, muscle weakness, and frailty (6–17). Several studies have demonstrated the link between GDF-15 and bone metabolism (19–21). However, the effect of GDF-15 as a myokine on bone metabolism and sarco-osteoporosis in humans is not clear. Hence, the aim of this study was to investigate the potential role of GDF-15 as a myokine in bone metabolism and muscle function in patients with osteoporosis.

The novel finding of the study was that GDF-15 may play a role in the inhibition of maintaining BMD and bone mass *via* endocrinological and paracrinological mechanisms. That role seemed to be more prominent in females with osteoporosis compared to healthy females. Both circulating and expression levels of GDF-15 in the pronator quadratus muscle were higher in osteoporotic patients than in controls. Furthermore, both serum concentration and mRNA expression levels of GDF-15 in muscle were negatively correlated with focal bone density, especially the cortical bone density of the distal radius, the attachment site of the pronator quadratus muscle as well as aBMD of the total hip, particularly in females with osteoporosis. According to several studies, the circulating level of GDF-15 is a negative predictor of BMD in post-menopausal females (32, 33) and other *in vitro* studies have demonstrated the influences of GDF-15 on osteoclastic activity (20, 21). This study supports the findings that circulating GDF-15 has a negative modulatory effect on maintaining BMD in the distal radius and total hip *via* endocrinological pathways. Moreover, the expression levels of GDF-15 in the pronator quadratus muscle that is attached to the distal radius appeared to be negatively related to maintaining BMD of the distal radius. Therefore, because levels of both circulating GDF-15 and expression GDF-15 in muscle were negatively related to the focal bone quality of the distal radius bone as well as aBMD of the total hip, we cautiously suggest that GDF-15 may have negative effects on bone mass maintenance *via* both endocrinological and paracrinological mechanisms, as a myokine that is released from the muscle.

The association between circulating GDF-15 levels and muscle mass and strength has been studied (6, 8). The findings from this study revealed patterns that differ from those found in previous reports. The circulating GDF-15 concentration did not correlate significantly with grip strength or circumferences of the upper arm and calf. Otherwise the mRNA expression levels of GDF-15 in the pronator quadratus muscle were significantly related to those variables. In one study, no significant correlations between circulating GDF-15 and muscle mass or strength were found in the elderly (34); another study that suggested plasma GDF-15 predicted sarcopenia-associated outcomes found no significant correlations between plasma GDF-15 levels and muscle mass or strength (12). The other study demonstrated that the expression levels of GDF-15 in muscle were negatively correlated with muscle weight and endurance capacity (10). We postulate that GDF-15 in muscle could be related to muscle mass and strength more significantly than is circulating GDF-15. We further postulate that this interaction might be more prominent in females with low bone mass.

TNF-α is a pro-inflammatory cytokine that is related to inflammation, mitochondrial dysfunction, and aging (35). TNF-α is involved in the pathogenesis of sarcopenia (36) and promotes pathological osteoclastogenesis and bone resorption in collaboration with receptor activator of nuclear factor kappa-B ligand (RANKL) (37). Our findings of negative correlations between circulating TNF-α levels and bone quality of the distal radius, circumference of calf, or grip strength are consistent with this phenomenon. Several studies have proposed that GDF-15 plays a role in the development of inflammation-driven states and in aging (13, 38), and one study suggested that GDF-15 may contribute to inflammatory damage through triglyceride metabolism (39). The finding that both circulating and mRNA expression levels of TNF-α in muscle were significantly correlated positively with those of GDF-15 could be understood in the same manner. The negative role of GDF-15 on maintaining bone mass could be related to the osteoclastic activity of TNF-α and the inflammation process that have negative effects on bone remodeling and muscle function.

This study primarily attempted to reveal the role of GDF-15 in bone and muscle metabolism in patients with osteoporosis. However, this study had several limitations. First, due to the small sample size, the statistical significance of the results is inevitably compromised. Second, because this study included only female participants, the information provided about the role of GDF-15 on bone metabolism and muscle function is limited. Third, because this study did not include data about skeletal muscle mass, degree of muscle fibrosis, and adipogenesis, the precise endocrinological mechanisms of GDF-15 could not be derived. Furthermore, the effects of aging on the circulation and expression levels of GDF-15 could not be ruled out because correlation analysis was not adjusted for age and other potential confounding factors due to multicollinearity among the variables. Finally, we measured only mRNA expression levels of the GDF-15 and inflammatory cytokines in muscle specimens, not protein levels, therefore the actual relationships between myokines and bone and muscle metabolism could not be assumed.

In conclusion, GDF-15 may have associations with bone metabolism in humans *via* both paracrinological and endocrinological pathways. Muscle mass and function would be influenced more prominent by GDF-15 mRNA expressed in muscle than by circulating GDF-15. The role of GDF-15 in bone metabolism and muscle homeostasis may be related to inflammatory responses. The findings of this study indicate that GDF-15 may be a target for future therapeutics for sarco-osteopenia and, eventually, for recovery and longevity. Future research is required.

## Data availability statement

The original contributions presented in the study are included in the article/**Supplementary Material**. Further inquiries can be directed to the corresponding author.

## Ethics statement

The studies involving human participants were reviewed and approved by Kangbuk Samsung Hospital. The patients/participants provided their written informed consent to participate in this study.

## Author contributions

All authors listed have made a substantial, direct, and intellectual contribution to the work and approved it for publication.

## Funding

This research was supported by the National Research Foundation of Korea (NRF) grant funded by the Korea government (No. 2020R1I1A1A01071537).

## Conflict of interest

The authors declare that the research was conducted in the absence of any commercial or financial relationships that could be construed as a potential conflict of interest.

## Publisher’s note

All claims expressed in this article are solely those of the authors and do not necessarily represent those of their affiliated organizations, or those of the publisher, the editors and the reviewers. Any product that may be evaluated in this article, or claim that may be made by its manufacturer, is not guaranteed or endorsed by the publisher.

## Supplementary material

Supplementary Figure Correlations between circulating levels of GDF-15 and **(A)** age, **(B)** aBMD of the total hip, **(C)** cortical bone density of the distal radius bone, **(D)** trabecular bone density of the distal radius bone. Correlations between extents of mRNA expression of GDF-15 and **(E)** age, **(F)** handgrip strength, **(G)** circumferences of upper arm, **(H)** circumferences of calf, **(I)** cortical bone density of the distal radius bone, **(J)** trabecular bone density of the distal radius bone, **(K)** serum concentration of the TNF-α, **(L)** mRNA expression levels of TNF-α.
