# Supplementary figures and images for: Growth differentiation factor-15 as a modulator of bone and muscle metabolism
Source: Front Endocrinol (Lausanne). 2022 Oct 17;13:948176. doi: 10.3389/fendo.2022.948176 (PMC9618662; doi:10.3389/fendo.2022.948176)

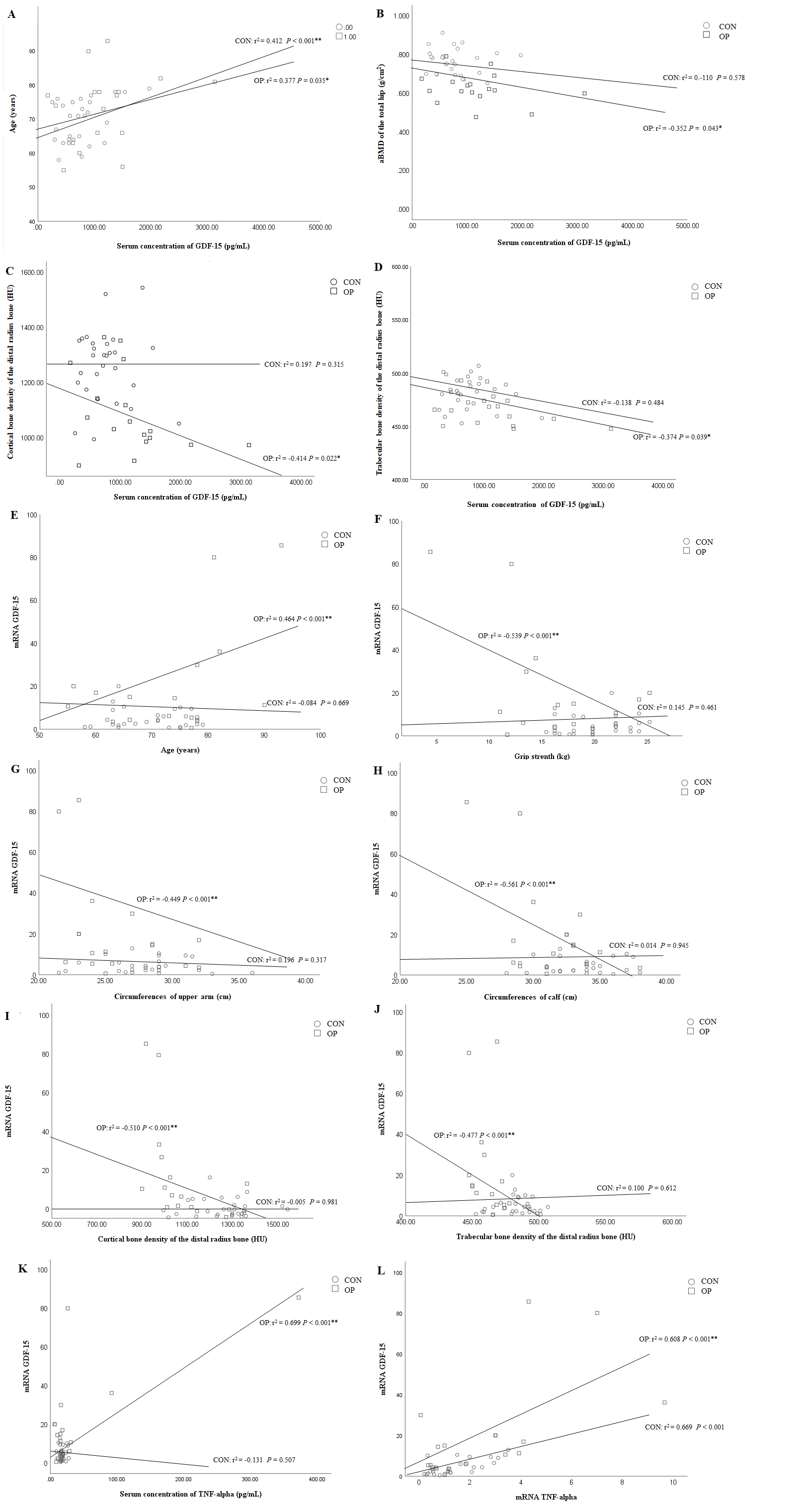

Supplement: Supplementary Figure — Correlations between circulating levels of GDF-15 and (A) age, (B) aBMD of the total hip, (C) cortical bone density of the distal radius bone, (D) trabecular bone density of the distal radius bone. Correlations between extents of mRNA expression of GDF-15 and (E) age, (F) handgrip strength, (G) circumferences of upper arm, (H) circumferences of calf, (I) cortical bone density of the distal radius bone, (J) trabecular bone density of the distal radius bone, (K) serum concentration of the TNF-α, (L) mRNA expression levels of TNF-α. [file Image_1.tif]
