# Supplementary material for: Growth differentiation factor-15 as a modulator of bone and muscle metabolism
Source: Front Endocrinol (Lausanne). 2022 Oct 17;13:948176. doi: 10.3389/fendo.2022.948176 (PMC9618662; doi:10.3389/fendo.2022.948176)
Supplement: Supplementary file 2 [file DataSheet_1.docx]

**Table 1.** Sequence each primer

| Primer | Forward primer | Reverse primer |
| --- | --- | --- |
| GDF-15 | CAATCCCATGGTGCTCATTC | TATGCAGTGGCAGTCTTTGG |
| IL-1β | GTACCTGTCCTGCGTGTTGA | GGGAACTGGGCAGACTCAAA |
| IL-6 | CTATGcAACTCCTTCTCCACAAGCGCCTT | GGGGCGGCTACATCTTTGGAATCTT |
| TNF-α | CTTCTGCCTGCTGCACTTTG | GTCACTCGGGGTTCGAGAAG |
| IGF-1 | CCATGTCCTCCTCGCATCTC | CGTGGCAGAGCTGGTGAAG |
| GAPDH | CCATCTTCCAGGAGCGAGATC | GCCTTCTCCATGGTGGTGAA |

**Table 2.** Differences of demographic features, BMD, and local radial forearm bone quality between two groups

|  | CON (n = 28) | OP (n = 17) | *P* value |
| --- | --- | --- | --- |
| Age (years) | 69.5 ± 6.3 | 73.4 ± 11.0 | 0.144 |
| Handgrip strength (kg) | 20.1 ± 2.9 | 16.4 ± 5.3 | 0.004* |
| BMI | 25.1 ± 3.0 | 23.2 ± 2.9 | 0.044* |
| Upper arm circumference | 28.9 ± 3.3 | 26.2 ± 3.4 | 0.103 |
| Calf circumference | 33.3 ± 2.6 | 31.6 ± 3.1 | 0.047* |
| Total hip aBMD (g/cm^2^) | 0.76 ± 0.07 | 0.62 ± 0.08 | < 0.001** |
| Total hip T score | -0.85 ± 0.70 | -2.12 ± 0.61 | < 0.001** |
| Femoral neck aBMD (g/cm^2^) | 0.64 ± 0.08 | 0.50 ± 0.04 | < 0.001** |
| Femoral neck T score | -1.70 ± 0.52 | -2.87 ± 0.39 | < 0.001** |
| L1-4 aBMD (g/cm^2^) | 0.84 ± 0.13 | 0.72 ± 0.12 | 0.004* |
| L1-4 T score | -1.64 ± 1.17 | -2.51 ± 0.96 | 0.010* |
| Total HU | 958.6 ± 131.0 | 854.2 ± 118.0 | < 0.001** |
| Cortical HU | 1261.2 ± 131.3 | 1087.4 ± 147.0 | < 0.001** |
| Cortical thickness (mm) | 1.21 ± 0.40 | 0.90 ± 0.21 | 0.005* |
| Trabecular HU | 481.8 ± 14.4 | 465.5 ± 14.1 | < 0.001** |

aBMD, areal bone mineral density; BMI, body mass index; L1-4, lumbar spine 1-4; HU, Hounsfield unit

Descriptive values are shown as mean ± SD.

Data obtained from independent T-test.

† Data obtained from Chi-square test.

** P* < 0.05, ** *P* < 0.001 by independent T-test and Chi-square test.

**Table 3.** Differences of serum levels of bone turnover markers, IGF-1, vitamin D, GDF-15, and inflammatory cytokines between CON and OP

|  | CON (n = 28) | OP (n = 17) | *P* value |
| --- | --- | --- | --- |
| CTX (ng/mL) | 0.45 ± 0.28 | 0.47 ± 0.31 | 0.904 |
| Osteocalcin (ng/mL) | 17.6 ± 9.1 | 22.6 ± 9.8 | 0.112 |
| IGF-1 (ng/mL) | 106.3 ± 50.2 | 102.7 ± 59.9 | 0.799 |
| 25-hydroxyvitamin D (ng/mL) | 25.4 ± 15.6 | 27.6 ± 15.5 | 0.788 |
| GDF-15 (pg/mL) | 831.0 ± 449.7 | 1167.0 ± 687.3 | 0.019* |
| TNF-α (pg/mL) | 18.6 ± 7.4 | 41.7 ± 84.6 | 0.115 |
| IL-6 (pg/mL) | 13.8 ± 14.1 | 16.8 ± 20.0 | 0.203 |
| IL-1 β (pg/mL) | 0.51 ± 0.24 | 0.56 ± 0.35 | 0.459 |

CTX, C-telopeptide of type I collagen; IGF-1, insulin-like growth factor-1; GDF-15, Growth differentiation factor-15, TNF-α, tumor necrosis factor-α; IL-6, interleukin-6; IL-1β, interleukin-1β

Descriptive values are shown as mean ± SD.

Data obtained from independent T-test.

** P* < 0.05, ** *P* < 0.001 by independent T-test.

**Table 4.** Correlation coefficient among age, hand grip strength, BMI, circumferences of upper arm and calf, aBMD of total hip, femoral neck, and lumbar spine, and local bone quality of radial forearm

|  | Age | Grip  strength | BMI | Upper arm circumference | Calf  circumference | Total hip  aBMD | Femoral  neck  aBMD | Lumbar  spine  aBMD | Total HU | Cortical HU | Cortical  thickness |
| --- | --- | --- | --- | --- | --- | --- | --- | --- | --- | --- | --- |
| Grip strength | **-0.579^**^** |  |  |  |  |  |  |  |  |  |  |
| BMI | -0.035 | 0.022 |  |  |  |  |  |  |  |  |  |
| Upper arm circumference | -0.201 | 0.249 | **0.624^**^** |  |  |  |  |  |  |  |  |
| Calf circumference | **-0.384^*^** | **0.388^*^** | 0.277 | 0.260 |  |  |  |  |  |  |  |
| Total hip aBMD | **-0.338^*^** | **0.452^**^** | 0.246 | 0.237 | **0.413^**^** |  |  |  |  |  |  |
| Femoral neck aBMD | -0.201 | **0.459^**^** | 0.148 | 0.122 | **0.329^*^** | **0.733^**^** |  |  |  |  |  |
| Lumbar spine aBMD | -0.137 | 0.190 | 0.200 | 0.096 | 0.188 | **0.357^*^** | **0.400^**^** |  |  |  |  |
| Total HU | **-0.364^*^** | 0.182 | 0.104 | 0.033 | -0.016 | **0.306^*^** | 0.167 | 0.281 |  |  |  |
| Cortical HU | **-0.300^*^** | **0.348^*^** | 0.144 | 0.148 | 0.185 | **0.462^**^** | **0.319^*^** | **0.323^*^** | **0.883^**^** |  |  |
| Cortical thickness | -0.141 | **0.315^*^** | 0.068 | 0.046 | 0.004 | **0.337^*^** | 0.214 | 0.185 | **0.701^**^** | **0.761^**^** |  |
| Trabecular  HU | **-0.302^*^** | 0.240 | 0.243 | 0.096 | **0.347^*^** | **0.432^**^** | **0.403^*^** | **0.418^**^** | **0.543^**^** | **0.654^**^** | **0.443^**^** |

BMI, body mass index; aBMD, areal bone mineral density; HU, Hounsfield units

^*^*P* < 0.05, ^**^*P* < 0.001 by Pearson’s correlation analysis

The figures in bold indicate values with statistical significance.

**Table 5.** Relationships among age, hand grip strength, BMI, circumferences of upper arm and calf, serum GDF-15 and cytokine levels, hand grip strength, aBMD of total hip, femoral neck, and lumbar spine, and local bone quality of radial forearm

|  | Serum  GDF-15 | Serum  TNF-α | Serum  IL-6 | Serum  IL-1 β | Serum  IGF-1 |
| --- | --- | --- | --- | --- | --- |
| Age | **0.374^*^** | **0.423^**^** | 0.150 | -0.071 | -0.247 |
| Grip strength | -0.227 | **-0.536^**^** | -0.190 | 0.040 | 0.152 |
| BMI | -0.234 | -0.105 | 0.063 | -0.001 | 0.235 |
| Upper arm circumference | -0.213 | -0.219 | 0.033 | -0.049 | **0.330^*^** |
| Calf circumference | -0.219 | **-0.438^**^** | -0.068 | 0.070 | 0.247 |
| Total hip aBMD | **-0.366^*^** | -0.291 | **-0.370^*^** | -0.103 | 0.212 |
| Femoral neck aBMD | -0.232 | -0.191 | **-0.335^*^** | -0.184 | -0.127 |
| Lumbar spine aBMD | -0.152 | -0.128 | -0.239 | -0.168 | 0.032 |
| Total HU | -0.248 | -0.125 | **-0.319^*^** | -0.139 | -0.036 |
| Cortical HU | **-0.354^*^** | **-0.317^*^** | **-0.357^*^** | -0.279 | 0.012 |
| Cortical thickness | -0.132 | -0.133 | **-0.406^**^** | -0.159 | -0.130 |
| Trabecular HU | **-0.373^*^** | -0.084 | -0.279 | **-0.305^*^** | 0.147 |
| Serum GDF-15 |  | 0.181 | -0.073 | 0.161 | -0.125 |
| Serum TNF-α |  |  | 0.035 | -0.165 | -0.132 |
| Serum IL-6 |  |  |  | **0.438^**^** | -0.094 |
| Serum IL-1 β |  |  |  |  | -0.084 |

BMI, body mass index; aBMD, areal bone mineral density; HU, Hounsfield units

^*^*P* < 0.05, ^**^*P* < 0.001 by Pearson’s correlation analysis

The figures in bold indicate values with statistical significance.

**Table 6.** Correlation among age, hand grip strength, BMI, circumferences of upper arm and calf, mRNA expression levels of GDF-15 and cytokine in muscles, aBMD of total hip, femoral neck, and lumbar spine, and local bone quality of radial forearm

|  | GDF-15 | TNF-α | IL-6 | IL-1 β | IGF-1 |
| --- | --- | --- | --- | --- | --- |
| Age | **0.404^**^** | 0.242 | 0.074 | **0.418^**^** | 0.212 |
| Grip strength | **-0.515^**^** | -0.192 | 0.114 | **-0.447^**^** | -0.177 |
| BMI | -0.182 | -0.182 | -0.214 | -0.238 | -0.179 |
| Serum GDF-15 | **0.564^**^** | **0.486^**^** | 0.202 | **0.511^**^** | 0.077 |
| Serum TNF-α | **0.694^**^** | **0.336^*^** | -0.105 | **0.625^**^** | 0.017 |
| Serum IL-6 | 0.033 | -0.056 | -0.087 | -0.065 | -0.099 |
| Serum IL-1 β | 0.125 | 0.075 | 0.058 | 0.062 | -0.112 |
| Serum IGF-1 | -0.144 | -0.051 | -0.128 | -0.186 | -0.102 |
| Upper arm circumference | **-0.378^*^** | **-0.309^*^** | 0.016 | **-0.366^*^** | -0.235 |
| Calf circumference | **-0.443^**^** | **-0.314^*^** | -0.225 | **-0.441^**^** | -0.292 |
| Total hip aBMD | **-0.392^*^** | **-0.426^**^** | 0.067 | -0.291 | -0.133 |
| Femoral neck aBMD | -0.248 | -0.267 | 0.044 | -0.198 | 0.043 |
| Lumbar spine aBMD | -0.276^*^ | -0.290 | -0.031 | -0.216 | 0.030 |
| Total HU | -0.209 | -0.050 | -0.078 | -0.163 | 0.016 |
| Cortical HU | **-0.480^**^** | -0.284 | -0.056 | **-0.402^**^** | 0.030 |
| Cortical thickness | -0.286 | -0.213 | -0.083 | -0.229 | 0.012 |
| Trabecular HU | **-0.374^*^** | **-0.351^*^** | -0.272 | **-0.396^*^** | 0.026 |
| GDF-15 |  | **0.643^**^** | -0.080 | **0.909^**^** | 0.140 |
| TNF-α |  |  | 0.001 | **0.639^**^** | 0.160 |
| IL-6 |  |  |  | 0.162 | 0.140 |
| IL-1 β |  |  |  |  | 0.140 |

BMI, body mass index; aBMD, areal bone mineral density; HU, Hounsfield units

^*^*P* < 0.05, ^**^*P* < 0.001 by Pearson’s correlation analysis

The figures in bold indicate values with statistical significance.
